# Dexterous manipulation: differential sensitivity of manipulation and grasp forces to task requirements

**DOI:** 10.1152/jn.00034.2024

**Published:** 2024-06-12

**Authors:** William P. Noll, Yen-Hsun Wu, Marco Santello

**Affiliations:** School of Biological and Health Systems Engineering, Arizona State University, Tempe, Arizona, United States

**Keywords:** dexterity, digits, grasping, sensorimotor control

## Abstract

How humans coordinate digit forces to perform dexterous manipulation is not well understood. This gap is due to the use of tasks devoid of dexterity requirements and/or the use of analytical techniques that cannot isolate the roles that digit forces play in preventing object slip and controlling object position and orientation (pose). In our recent work, we used a dexterous manipulation task and decomposed digit forces into *F*_G_, the internal force that prevents object slip, and *F*_M_, the force responsible for object pose control. Unlike *F*_G_, *F*_M_ was modulated from object lift onset to hold, suggesting their different sensitivity to sensory feedback acquired during object lift. However, the extent to which *F*_G_ and *F*_M_ can be controlled independently remains to be determined. Importantly, how *F*_G_ and *F*_M_ change as a function of object property is mathematically indeterminate and therefore requires active modulation. To address this gap, we systematically changed either object mass or external torque. The *F*_M_ normal component responsible for object orientation control was modulated to changes in object torque but not mass. In contrast, *F*_G_ was distinctly modulated to changes in object mass and torque. These findings point to a differential sensitivity of *F*_G_ and *F*_M_ to task requirements and provide novel insights into the neural control of dexterous manipulation. Importantly, our results indicate that the proposed digit force decomposition has the potential to capture important differences in how sensory inputs are processed and integrated to simultaneously ensure grasp stability and dexterous object pose control.

**NEW & NOTEWORTHY** Successful dexterous object manipulation requires simultaneous prevention of object slip and object pose control. How these two task goals are attained can be investigated by decomposing digit forces into grasp and manipulation forces, respectively. We found that these forces were characterized by differential sensitivity to changes in object properties (mass and torque). This finding suggests the involvement of distinct sensorimotor mechanisms that, combined, simultaneously ensure grasp stability and dexterous control of object pose.

## Graphical abstract

**Figure:**
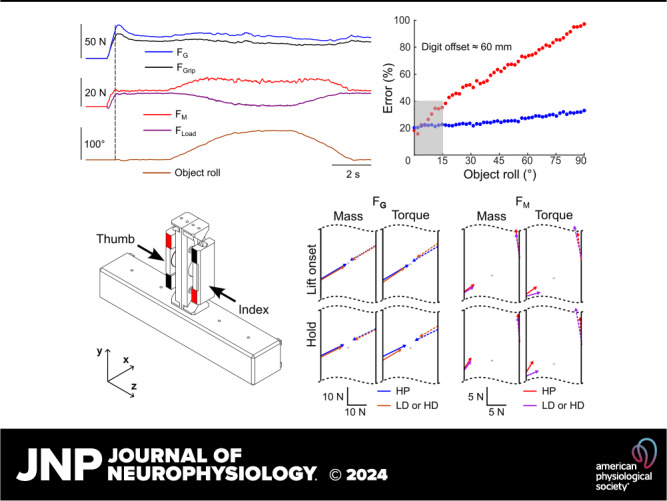

## INTRODUCTION

Human dexterity relies on complex sensorimotor interactions. For instance, when we reach and grasp for a glass of water to drink, digit forces must be coordinated to tilt the glass while preventing it from slipping. Humans’ exquisite ability to perform dexterous manipulation has been extensively studied over the past four decades across multiple dimensions. These dimensions range from kinematics, i.e., the spatial and temporal coordination of reaching and grasping (1–4) and choice of contact points (5–10), kinetics, i.e., the development of digit forces from contact to the onset of manipulation (11–13), and the transition from kinematics to kinetics, i.e., the coordination of digit forces as a function of fingertip position (14–16). Significant insights have also been provided by research addressing central and peripheral neural mechanisms underlying sensorimotor control of dexterous manipulation (for review, see Refs. 17–19).

A critically important feature of successful manipulation is the ability to coordinate digit forces to simultaneously prevent object slip while changing object position and orientation (pose). Surprisingly, how humans accomplish these two task goals has been largely unexplored. As pointed out in our recent study (20), this gap is partly due to the use of tasks that do not have significant dexterity requirements and partly due to the use of analytical techniques that cannot isolate the two roles that digit forces play in preventing object slip and controlling object pose.

**Figure 1.**
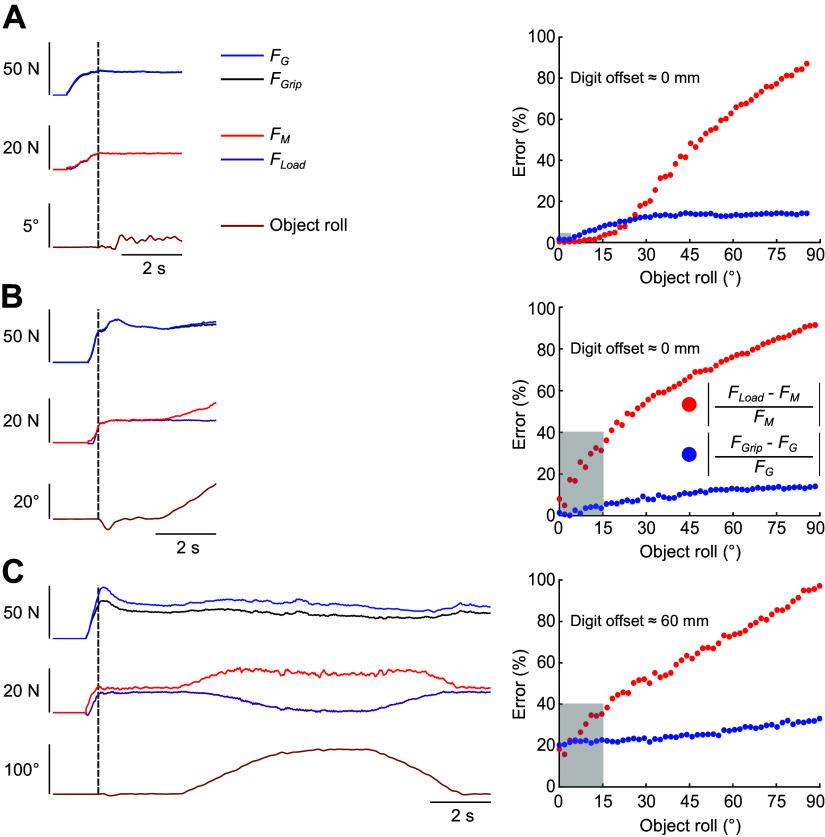
Comparison of methods for analyzing digit forces during grasping and manipulation. The left column plots show the time course of digit forces and object roll, whereas the right column plots show the comparison between the traditional digit force analysis and the analysis based on the manipulation and grasp force decomposition algorithm (MGFD). Plots on the left column show grip and load forces (*F*_Grip_ and *F*_Load_, respectively) obtained through the traditional digit force analysis, grasp and manipulation forces (*F*_G_ and *F*_M_, respectively) obtained through the MGFD, and object roll. The dashed vertical lines denote object lift onset. Plots on the right column show the error (%) caused by using the traditional force analysis relative to the MGFD for each task context. The formula used to compute the estimation error is shown in the legend. The shaded gray region denotes the error based on the task context and object roll. *A*: example data from one subject grasping and manipulating an object with a symmetrical mass distribution and collinear contacts (digit offset = ∼0 mm). This setup was pioneered by Westling and Johansson in the 1980s (21) and colleagues to study digit force coordination during object grasping and lifting. *B*: example data from the same subject grasping and manipulating an object with an asymmetrical mass distribution (inverted T-object shape) and collinear contacts (digit offset = ∼0 mm). This setup was first introduced by Gordon and colleagues (22) to study digit force coordination during object grasping and lifting while minimizing the object roll caused by the object’s asymmetric mass distribution. *C*: example data from the same subject grasping and manipulating an object with an asymmetrical mass distribution (inverted T-object shape) and noncollinear contacts (digit offset = ∼60 mm). This setup was first introduced by Santello and colleagues (15) to study the coordination of digit forces and positions when contacts are not predetermined by the experimenter (“unconstrained grasping”).

To address the aforementioned gaps, we have previously proposed an approach based on manipulation and grasp force decomposition (MGFD) together with a task that requires dexterous minimization of object roll (20). Briefly, MGFD decomposes digit forces into grasp force (*F*_G_), the force required to prevent object slip, and manipulation force (*F*_M_), the force required to control object pose. Figure 1 shows three manipulation task contexts and the limitations of traditional digit force analysis. Grip and load forces (21) are correctly defined as being exerted perpendicular and tangential to the contact surface, respectively, by the digits positioned at zero digit offset (defined as the vertical distance between contacts) when the object’s mass is symmetrically distributed and the object does not roll during manipulation (Fig. 1*A*, left plot). We will refer to the analysis of grip and load forces as defined earlier as “traditional digit force analysis.” In this task context, load forces are used to support object weight, whereas grip force is used to prevent object slip. Furthermore, pose control, which is accomplished by load forces, is limited to lifting and maintaining object position as no object orientation control is required. For this task scenario, the output of traditional and MGFD analysis of digit forces match, as shown by overlapping force traces.

However, when digit offset is approximately zero and the object’s mass is not symmetrically distributed, the external torque induced by the asymmetrically distributed mass will cause the object to roll during manipulation (Fig. 1*B*, left plot). The task now requires subjects to coordinate digit forces to not only prevent object slip but also minimize object roll. When the object rolls, a portion of grip force now must also contribute to supporting object weight, while the digits have to exert asymmetrical load forces to control object pose. In this task context, digit force estimates by the traditional and MGFD analyses diverge when the object rolls. A third scenario consists of grasping and manipulation of an object with asymmetrical mass distribution and at nonzero digit offsets (Fig. 1*C*, left plot). Here, force estimates by the two analyses diverge to a greater extent than for the task context shown in Fig. 1*B* even in the absence of object roll.

A direct comparison between the performance of traditional digit force analysis and MGFD is shown in the right column of Fig. 1. When the object’s mass is symmetrically distributed, digit offset is close to zero, and there is no or negligible object roll, the two techniques provide the same results (shaded area in Fig. 1*A*, right plot). However, the estimation of digit forces by the two techniques significantly diverges (up to 35% error) when the object rolls (∼15°, as in Ref. 22) due to the asymmetric object’s mass distribution (shaded area in Fig. 1*B*, right plot). Even greater force estimation errors occur when applying the traditional force analysis to grasping and manipulation of an object at nonzero digit offsets (15, 23) (shaded area in Fig. 1*C*, right plot). Here, a significant divergence in the output of the two techniques (∼20%) occurs even at 0° object roll and increases monotonically with increasing object roll.

Based on the aforementioned considerations, *F*_G_ and *F*_M_ provide a much clearer separation of digit forces based on their distinct functional roles regardless of object pose and digit offset relative to the traditional digit force analysis. Importantly, MGFD allows the separation of biomechanically obligatory versus nonobligatory modulation of *F*_G_ and *F*_M_. Regarding the biomechanically nonobligatory modulation, how *F*_G_ and *F*_M_ change as a function of object property is mathematically indeterminate because the number of grasp variables (3 forces per digit + 1 moment of force per digit + 1 two-dimensional contact location, i.e., digit offset) is greater than grasp constraints (3 position + 3 rotation constraints). By applying MGFD, we recently reported that humans prioritize grasp stability over efficiency when modulating *F*_G_ across grip configurations (20). Furthermore, little to no modulation of *F*_G_ occurred from object lift onset to hold, whereas *F*_M_ underwent significant modulation across these two task epochs. We interpreted these results as evidence for the interplay of distinct sensorimotor processes, such that *F*_G_ and *F*_M_ would be primarily mediated by feedforward and feedback control mechanisms, respectively. These findings, together with the results from another study showing distinct adaptation processes for grasp and manipulation torques (24), prompted the question of whether humans can control *F*_G_ and *F*_M_ independently.

The goal of the present study was to test this theoretical framework by systematically changing object properties, mass or external torque, to selectively challenge object slip prevention or pose control, respectively. We reasoned that if the central nervous system (CNS) can control *F*_G_ and *F*_M_ independently, challenging object slip prevention by increasing object mass only should lead to a modulation of *F*_G_ but not *F*_M_, as the latter would be sensitive only to challenges in object pose control. Conversely, increasing object torque should cause a modulation of *F*_M_ but not *F*_G_, as the latter would respond only to increasing risk of object slip. However, modulation of *F*_G_ and/or *F*_M_ to changes in both object mass and torque would be interpreted as evidence for low or no independent control of these two functionally distinct forces. Based on our recent findings of distinct sensorimotor control mechanisms (20), we hypothesized that subjects would selectively modulate *F*_G_ and *F*_M_ to changes in object mass and torque, respectively.

## METHODS

### Experimental Design

**Figure 2.**
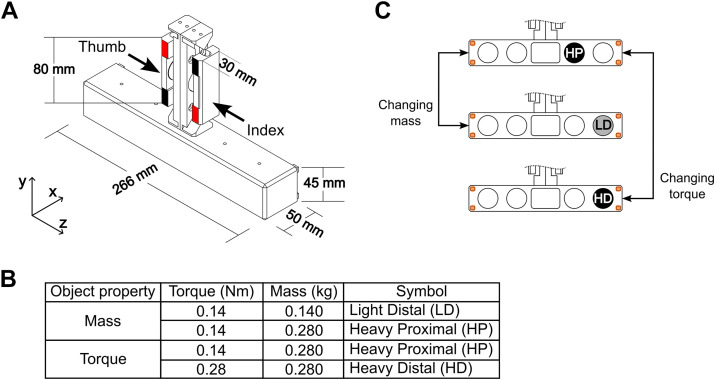
Grip device and experimental conditions. *A*: the grip device consists of a custom-made vertical handle equipped with force/torque sensors mounted on a horizontal base. Red and black tapes were used to cue subjects on where to place the thumb and index finger according to the experimental condition (left or right center of mass, L or R CM, respectively). White tape was used to cue subjects’ digit placement when estimating the coefficient of friction. *B*: combinations of object torque and mass used for each experimental condition. *C*: examples of left center of mass (L CM) experimental contrasts: light vs. heavy mass (LD-HP) and proximal vs. distal (relative to the geometric center of the handle) torque (HP-HD). The four orange blubs denote infrared LED markers attached to the four outer corners of the horizontal base used for motion tracking.

We asked participants to grasp an instrumented inverted T-shaped object (Fig. 2*A*) using the thumb and index fingertip, lift the object while preventing it from tilting, hold it, and return it to its starting location on the table. To emphasize the dexterous component of the task, we instructed participants to lift the object as if it were a glass filled with water and to prevent any spillage. To minimize object tilt, subjects had to generate a compensatory torque of the same magnitude and in the opposite direction of the external torque in an anticipatory fashion, i.e., at object lift onset (15). We systematically changed object properties (mass or external torque; Fig. 2*B*) to investigate the coordination of digit forces as a function of object properties. We note that our task requires simultaneous object slip prevention and pose control. As done in our previous study (20), we used a manipulation and grasp force decomposition (MGFD) algorithm to identify grasp, manipulation forces, and the relation between them.

### Participants

We recruited 20 right-handed (self-reported) adults (10 males and 10 females; average age ± SD: 20.9 ± 1.8 yr). All study participants had normal to corrected vision and no history of neurological disorders. All individuals were naïve to the purpose of the study and provided informed written consent in accordance with the Declaration of Helsinki, however, the study was not registered in a public database. The experimental protocols were approved by the Office of Research Integrity and Assurance at Arizona State University.

### Apparatus

We asked subjects to grasp and manipulate a custom-made grip device with the thumb and index finger. The device consists of a custom-made vertical handle with two graspable surfaces (one for the thumb and one for the index finger) connected to a horizontal base (Fig. 2*A*). Each graspable surface was instrumented with one six-component force/torque (F/T) transducer (Nano 25, ATI Industrial Automation, Garner, NC). The transducers measure forces and moments of forces exerted by each digit on the graspable surfaces (sampling rate: 1 kHz). The position and orientation of the object and hand were tracked by a 10-camera active infrared marker motion capture system (Impulse, PhaseSpace Inc., San Leandro, CA; sampling rate: 480 Hz). We used four infrared LED markers on the object (Fig. 2*C*). A customized LabVIEW program (National Instruments, Austin, TX) was used to stream kinetic and kinematic data to the hard drive of the host computer.

The horizontal base of the device has five compartments: one is in the center of the base, and two are located 50 and 100 mm to the left and the right of the center location, respectively (Fig. 2*C*). A mass (0.140 or 0.280 kg) was added to one of these compartments to increase the object’s mass and/or an external torque. The cables of the F/T sensors were suspended to prevent them from generating torque on the object. The total mass of the object with no added mass was 0.8 kg. To cue subjects where to grasp the vertical handle within each experimental condition, we placed tape with different colors on the frontal plane of the two parallel graspable surfaces: the areas denoted by red and black tape denoted the grasp location for the left (L) and right (R) center of mass (CM) experimental conditions, respectively (Fig. 2*A*).

### Experimental Protocol

The goal of the present study was to determine the extent to which grasp and manipulation forces (*F*_G_ and *F*_M_, respectively) can be modulated independently as a function of object properties. Specifically, *F*_G_ is responsible for object slip prevention, whereas *F*_M_ is responsible for the control of object position and orientation (pose) (20). Therefore, we systematically changed *1*) object mass to determine whether subjects would selectively modulate *F*_G_ to minimize the risk of object slip and *2*) object external torque in the grasp plane, i.e., the *yz* plane in the object coordinate system (Fig. 2*A*), to determine whether subjects would selectively modulate *F*_M_ to accurately control object pose.

To determine participants’ modulation of *F*_G_, we changed the task requirement of object slip prevention by using two mass conditions, heavy or light mass, while keeping the requirement for object pose control (external torque) constant. This objective was attained by adding a 280 g mass at 50 mm (heavy mass, H) or a 140 g mass at 100 mm (light mass, L) from the center of the object’s horizontal base (Fig. 2, *B* and *C*). For both H and L conditions, the external torque (*T*_EXT_) on the object was 140 N·mm. To determine participants’ modulation of *F*_M_, we changed the task requirement of object pose control by using two external torque conditions while keeping the object slip requirements (object mass) constant. These conditions consisted of adding a 280 g mass at 50 mm (proximal mass position, P) or 100 mm (distal mass position, D) from the center of the object’s horizontal base (Fig. 2, *B* and *C*). The P and D conditions resulted in 140 N mm and 280 N mm *T*_EXT_ on the object, respectively. Therefore, we tested three experimental conditions within the left (L) and right (R) CM conditions for a total of six conditions: variable object mass and constant torque (left CM: LHP and LLD; right CM: RHP and RLD) and variable external torque and constant object mass (left CM: LHP and LHD; right CM: RHP and RHD). The combinations of mass and external torque locations were chosen based on pilot tests and the extent to which participants could perform our dexterous manipulation task comfortably. We tested our experimental conditions for L and R CM to determine the extent to which the relation between *F*_G_ and *F*_M_ is independent of external torque direction.

To ensure a natural grasp configuration, grasping and manipulation of the object with the L or R CM require the thumb to be positioned higher or lower than the index finger, respectively. As the location of the added mass was hidden from view, participants were cued about where to position the thumb relative to the index finger by color-coded tape on the graspable surfaces (Fig. 2*A*). The length of each tape was sufficiently large (23 mm) to enable participants comfortable and natural positioning of the digits. For the L CM conditions (LHP-LLD and LHP-LHD), red tape cued participants to place the thumb higher than the index fingertip, whereas for the R CM conditions (RHP-RLD and RHP-RHD), black tape cued participants to place their thumb lower than the index fingertip. An additional reason for cueing digit placement and the external torque direction was to prevent subjects from positioning their thumb and index fingertips at very short vertical distances. This would have resulted in a shorter moment arm for digit normal forces, thereby causing digit tangential forces to become the primary contributor to the compensatory torque (e.g., see Ref. 22 and unconstrained condition in Ref. 20, see *Eq. 2*). White tape (Fig. 2*A*) was used to cue subjects where to position their fingertips for trials used to estimate the coefficient of friction (see *Data Processing*).

The object was placed 30 cm in front of the participant on a tabletop 30 cm below the participant’s shoulder joint. The first auditory cue (READY) prompted the subject to prepare for the reach. Next, participants were instructed to start reaching for the object after hearing the auditory cue (GO), grasp it using a grip configuration denoted by the color-coded tape, and lift it straight at a self-paced speed. To enforce the dexterity requirement of object pose control, participants were encouraged to pretend the handle was a glass full of water and lift it while minimizing spillage. Participants were instructed to lift the object at approximately chin height. When the object reached a minimum height threshold of 20 cm above the table, a third auditory cue (HOLD) informed participants to start holding the object stationary and maintaining the pose for ∼2 s. The next auditory cue (RELAX) informed participants to replace the object on the table and move the hand back to the initial position. Subjects were given ∼10-s breaks between trials and ∼60-s breaks between blocks to minimize fatigue.

To familiarize participants with the task and protocol, three trials were performed with no added mass to the object. These trials were followed by 20 consecutive trials for each of the six experimental conditions (HP, LD, HD for L and R CM), resulting in a total of 120 trials. All experimental conditions were presented in blocks of 20 consecutive trials to give subjects ample time to attain stable performance, i.e., object roll minimization.

Half of the participants in this study also participated in an experiment where object mass and external torque were changed in a random fashion across trials (“object property randomization”) before performing the current study. To avoid carry-over effects, these participants were tested in the current study after at least 1 wk from the “object property randomization” study session. A linear mixed-effect model on all dependent variables was performed to compare participants who performed the random condition before the current study versus participants who performed the current study before the random condition. This analysis revealed no significant difference in any of the experimental variables (*t*_16.34_ = 1.87, *P* > 0.05).

### Data Processing

Kinematic and kinetic data were resampled offline to 250 Hz, temporally aligned, and processed for analysis by custom software written in MATLAB (MathWorks Inc., Natick, MA). All data were first low-pass filtered at 30 Hz with second-order, zero-lag Butterworth filters.

Digit forces were analyzed at two task epochs: object lift onset and object hold. As done in our previous work (20), our analyses focused on these two epochs to contrast anticipatory control of digit forces before acquiring sensory feedback about object properties (object lift onset) versus steady-state digit force control (object hold) after participants experienced the dynamics of the object throughout object lift. Object lift onset was computed by finding the time at which object velocity reached its maximum value, and then searching backward for the first time point at which the object height was less than 1 mm with a velocity less than 5 mm/s. Object hold onset and offset were defined as 500 ms and 1,500 ms after the auditory hold cue, respectively. To verify that this definition of object hold onset did not overlap with the transient force fluctuations occurring toward the end of object lift, we performed the following analysis. We first determined the end of object lift by searching forward from the object’s peak velocity for the last time point at which the object velocity was less than 76 mm/s (10% of maximum object velocity across all trials and subjects). We then normalized variables during the lift duration (from lift onset to the end of object lift) across trials and subjects. The median (±SE) end of object lift was 320 ms (±28 ms) after the auditory cue, thus indicating that the period between hold onset and offset captured the static object hold epoch.

### Data Analysis

#### Digit force decomposition into grasp and manipulation forces.

We decomposed digit forces into grasp and manipulation forces as described by Wu and Santello (20). Briefly, each digit force vector is assumed to be applied at its point of application (center of pressure, CoP). These contact forces and the moment of force exerted by each digit at the contact surface in the object-center coordinate frame can be represented as a generalized force vector, ${\vec{F}}_{\text{C}}$. To transform these forces to the origin of the object-center coordinate frame, an overall grasp map (*G*) can then be written based on the force application locations of each digit using the soft finger contact model assumption (25). The grasp force (*F*_G_) is responsible for preventing object slip and is defined as the internal force that lies in the null space of *G* and that does not cause any motion of the object. The manipulation force required to control object pose (*F*_M_) is computed as the total finger force minus *F*_G_ (*Eq. 1*):

(*1*) $${\vec{F}}_{\text{M}}={\left[\begin{array}{cc} {\vec{F}}_{\text{C}}^{\text{TH}} & {\vec{F}}_{\text{C}}^{\text{IN}} \end{array}\right]}^{T}-{\vec{F}}_{\text{G}}$$

where TH and IN denote thumb and index finger, respectively. We note that the digit force decomposition approach based on the grasp map has been used in previous studies to identify internal forces (grasp forces, *F*_G_) exerted during object manipulation (26, 27). Specifically, Gao et al. (26) computed internal force and internal moment of force from a simplified grasp map based on the virtual-finger assumption (28), whereas Singh and Ambike (27) estimated the tightness of the grip from the internal force extracted from the grasp map. However, none of these studies quantified the minimum grasp force required to prevent object slip ($F_{\text{G}}^{\min}$). Analysis of $F_{\text{G}}^{\min}$ (described later) is important to separate biomechanically obligatory from nonobligatory effects of a given experimental factor, e.g., grasp configuration (20) or object properties (present study), on *F*_M_ and *F*_G_ modulation.

We note that *F*_M_ will always be equal and opposite to gravity at static equilibrium. However, the variables in the grasping system consist of a four-dimensional force vector (3 forces and 1 moment) per digit and a two-dimensional coordinate (*x*- and *y*-digit offset, i.e., the difference in *x*- and *y*-coordinates of thumb and index fingertip center of pressure), hence *m* = 10 variables across the two digits. However, gravity imposes only *n* = 6 constraints (3 translational and 3 rotational) in the three-dimensional (3-D) space. As a result, the solution to attain equilibrium is not unique, i.e., it is mathematically indeterminate because the number of variables (*m* = 10) is greater than the number of constraints (*n* = 6). Even though our analysis of *F*_M_ components considered only the *yz* plane dynamics (see frame of reference in Fig. 2*A*), the number of variables (*m* = 5: *z-* and *y*-force components per digit, and *y*-digit offset) is still greater than the number of constraints (*n* = 3, i.e., *z*- and *y*-translation, rotation in the *yz* plane). Therefore, the greater number of variables than constraints requires active modulation of *F*_G_ and *F*_M_ to perform our manipulation task.

#### Coefficient of friction estimation.

We estimated the coefficient of friction between the fingertips and the graspable surfaces from the slip force measurement. The translational coefficient of friction was defined as the ratio between the minimal finger force normal to the graspable surface required to prevent slip and the tangential finger force measured at the object slip onset (21). As this method requires fingertip tangential (vertical) forces to be equal and aligned with the gravitational force, we asked participants to grasp the object with no added weight and, therefore, no external torque at collinear contacts denoted by white tape on the graspable surfaces (Fig. 2*A*). We instructed participants to hold the object 5 cm above the table and “slowly move the index finger and thumb apart and let the object drop freely when it slips.” To assess the extent to which the coefficient of friction might have changed throughout the experimental session, we compared three object release trials at the beginning and end of the experimental session.

#### Minimum and excessive grasp force.

We estimated the minimal *F*_G_ required to prevent object slip, $F_{\text{G}}^{\min}$, using an optimization process as described by Wu and Santello (20). Briefly, we used *F*_M_, the grasp map, and the coefficient of friction (see aforementioned) to minimize the magnitude of *F*_G_ with translational and torsional friction cone constraints (29). The magnitude of $F_{\text{G}}^{\min}$ was computed as $|F_{\text{G}}^{\text{min}}|\;=\;\sum_{i\;=\;\text{TH, IN}}\left|{\vec{F}}_{\text{G}}^{\text{min},\;i}\right|$. The excessive grasp force, $F_{\text{G}}^{\text{EX}}$, denoting *F*_G_ chosen by the subject above the mechanically obligatory $F_{\text{G}}^{\min}$, was defined as the difference between the magnitude of *F*_G_ and $F_{\text{G}}^{\min}$, i.e., $F_{\text{G}}^{\text{EX}}$ = |*F*_G_| − |$F_{\text{G}}^{\min}$|.

#### Compensatory torque.

In our previous work (20), we adapted an earlier definition of compensatory torque (*T*_COM_; 15) to denote the resultant torque that the subject exerts on the object to counteract the external torque caused by the object’s asymmetrical mass distribution. Here, we define *T*_COM_ using *F*_M_ components as follows:

(*2*) TCOM = ry×ΔFMy+rz×ΔFMz

where *^y^r* and *^z^r* are the moment arms of the difference between thumb and index finger tangential (*^y^F*_M_) and normal (*^z^F*_M_) *F*_M_ components, respectively. We analyzed *T*_COM_ to determine the trial after which subjects attained a stable performance.

#### Coefficient of variation of F_M_ and F_G_.

To assess the across-trial variability of $F_{\text{G}}^{\text{EX}}$ and *F*_G_ while accounting for their different magnitudes (e.g., Fig. 4), we computed the coefficient of variation (CV) of these variables across *trials 4–20* for each subject and experimental condition. As done in our previous work (20), before statistical analysis we normalized the CV at lift onset by the CV during object hold for both $F_{\text{G}}^{\text{EX}}$ and *F*_M_.

### Statistical Analysis

Statistical analyses consisted of linear mixed-effect models (LMMs) and circular mixed-effects models (CMMs) of magnitudes and angles of *F*_G_ and *F*_M_ force vectors, respectively. These analyses were performed in RStudio (v.1.3.1093) using R (v.4.3.0) at the *P* < 0.01 significance level for LMMs and 95% highest posterior density intervals for CMMs. LMMs were used to compare the relations between *F*_G_ and *F*_M_ as a function of a combination of object properties (mass and external torque), whereas CMMs addressed the effects of object properties on force vector directions. For LMMs, the “lmerTest” package was used to fit all dependent variables with a restricted maximum likelihood criterion. The degrees of freedom for the corresponding *P* values were approximated for all LMMs with Satterthwaite’s method. We used the “bpnreg” package (30, 31) to fit Bayesian CMMs for angles of force vectors.

#### Linear mixed-effects models.

To examine the slopes of kinetic variables among changing mass and changing torque conditions, three-way linear mixed-effects models (LMMs) were created starting with fixed effects of Epoch (2 levels: Lift, Hold), CM (L, R), and Object Property (3 levels: HP, LD, HD). Hold, R CM, and HP were used as the fixed effect baselines. All models were built with random slopes and intercepts for the random-effect factor Subject accounting for differences in hand size, dexterity, as well as other between-subject differences. The procedure for fitting the models began by determining the appropriate random slopes and intercepts. To do this, we fitted models with various combinations of random slopes and intercepts while all fixed effects were present in the model. The best-fitting random effects were determined based on the model with the highest log-likelihood (32). Using these random effects, we ran a model with all fixed effects. When the fixed effects of Epoch and CM were above the statistically significant threshold of *P* = 0.01 or had minuscule estimates, they were removed from the model and the model was run again (32). This process was repeated individually for each dependent variable. When investigating *F*_M_ and its components, we found that CM was statistically significant, with an estimate of roughly half the size of the object property fixed effect. However, as the effects of CM were not the primary focus of the present study, and CM as a fixed effect (included or removed from the model) did not affect the results of object property, CM was removed from the final model.

#### Circular mixed-effects models.

To quantify the effects of object property and task epoch on the angle of *F*_G_ and *F*_M_ vectors, we first computed the vectorial mean and angular deviation (33) across trials and subjects per experimental condition and task epoch. Angles were computed in radians using the MATLAB function “atan2” and inputting the force vector’s *y* and *z*-component. To determine statistical significance, 95% highest posterior density intervals computed from CMMs were used to determine if the force vector angles were different (i.e., nonoverlapping). Each model had the fixed effects of epoch (2 levels: Lift, Hold) and object property (3 levels: HP, LD, HD). Hold and HP were used as the fixed effect baselines. Separate models were run for L and R CMs. All models were built with random intercepts for the random-effect factor Subject. For each model, we ran 500 iterations, with the first 50 iterations set to discard. This procedure was performed to ensure the model reached a stable state and accurately sampled from the true distribution of interest while avoiding overfitting. A lag of 1 was set to prevent autocorrelation between the parameter estimates.

## RESULTS

Participants performed a dexterous manipulation task of a sensorized object using a precision grip. We changed the properties of the object in a blocked fashion by changing either its mass (while keeping the external torque constant) or its external torque in the grasp plane, i.e., the *yz* plane in the object coordinate system (while keeping the mass constant) (Fig. 2*A*). The rationale for this study design was to assess the extent to which grasp and manipulation forces (*F*_G_ and *F*_M_)—required to simultaneously prevent object slip and control object pose, respectively—would be selectively modulated to changes in either object mass or torque. *F*_G_ and *F*_M_ were computed using a manipulation and grasp force decomposition (MGFD) algorithm (see methods).

We first briefly report the results of the analysis of across-trial variability of *F*_G_ and *F*_M_ to determine whether the present data would confirm previous findings on differences in the underlying sensorimotor control mechanisms (20), followed by the analysis of the performance of our manipulation task and the sensitivity of *F*_G_ and *F*_M_ modulation as a function of object properties.

### Modulation of Grasp and Manipulation Forces at Object Lift Onset versus Hold

We recently reported that *F*_G_ and *F*_M_ were characterized by distinct modulation patterns to changes in grip configuration when comparing object lift onset versus hold (20). Specifically, we found that *F*_G_ was statistically indistinguishable across these two task phases. In contrast, *F*_M_ significantly changed when transitioning from object lift onset to hold. We interpreted these results as evidence for distinct sensorimotor control mechanisms: *F*_G_ is primarily controlled in a feedforward fashion, whereas *F*_M_ is primarily mediated by feedback control. Analysis of across-trial variability of *F*_G_ and *F*_M_ at object lift onset and hold pooled across all experimental conditions confirmed our previous findings (see Supplemental Fig. S1).

### Performance of Dexterous Manipulation: Compensatory Torque and Peak Object Roll

**Figure 3.**
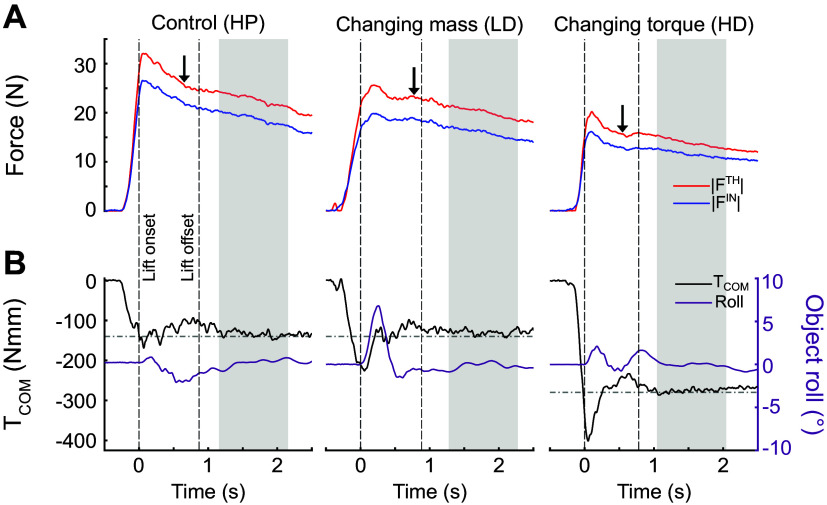
Performance of dexterous manipulation: digit forces, compensatory torque, and object roll*. A*: time course of thumb and index fingertip forces (red and blue lines, respectively). *B*: metrics of dexterous manipulation performance (compensatory torque, *T*_COM_, and object roll, denoted by black and purple lines, respectively). Data are from one subject (S13) and *trial 17* for each condition. Left and right dashed vertical lines denote object lift onset and end, respectively. The arrow highlights the “Hold” audio cue. Horizontal dashed-dotted lines denote the target *T*_COM_ required to prevent object roll. Gray boxes denote the epoch used to analyze data during object hold. Data in each column are from the heavy mass, proximal mass position (HP; left column), the light mass, distal mass position (LD; center column), and heavy mass, distal mass position (HD; right column) experimental conditions. Note that *1*) the HD condition is characterized by the same mass but a larger external torque than the HP condition, and *2*) the HP condition is characterized by the same external torque but a larger mass than the LD condition.

We quantified the extent to which participants complied with our task instructions by measuring compensatory torque at object lift onset (*T*_COM_) and peak object roll during the lift (see methods). Figure 3 shows representative trials of the same subject performing our task in three experimental conditions (heavy mass, proximal mass position: HP; light mass, distal mass position: LD; heavy mass, distal mass position: HD). Figure 3*A* shows the time course of thumb and index fingertip contact force magnitudes, whereas Fig. 3*B* shows the time course of *T*_COM_ and object roll.

Visual comparison of digit forces shows that, although the time course of digit forces was similar across experimental conditions, this participant exerted larger forces for the HP than LD and HD conditions during object lift (epoch within the dashed vertical lines) and hold (gray shaded area). As expected from our previous work, after this participant had learned to counter the external torque following a few object lifts, *T*_COM_ started to increase ∼250 ms before object lift onset and approached the expected external torque (horizontal dashed-dotted line) at lift onset across all experimental conditions (Fig. 3*B*). Consequently, peak object roll was relatively small (∼2° to 4°).

The data shown in Fig. 3 were representative of all subjects and replicated results from our previous work. Specifically, linear mixed models (LMMs) performed on peak object roll and *T*_COM_ with trial number as a fixed effect and random slopes revealed no significant differences from trials 4 through 20 (largest *t* statistic: *t*_252.14_ = −0.76, all *P* values > 0.10).

### Grasp and Manipulation Forces

**Figure 4.**
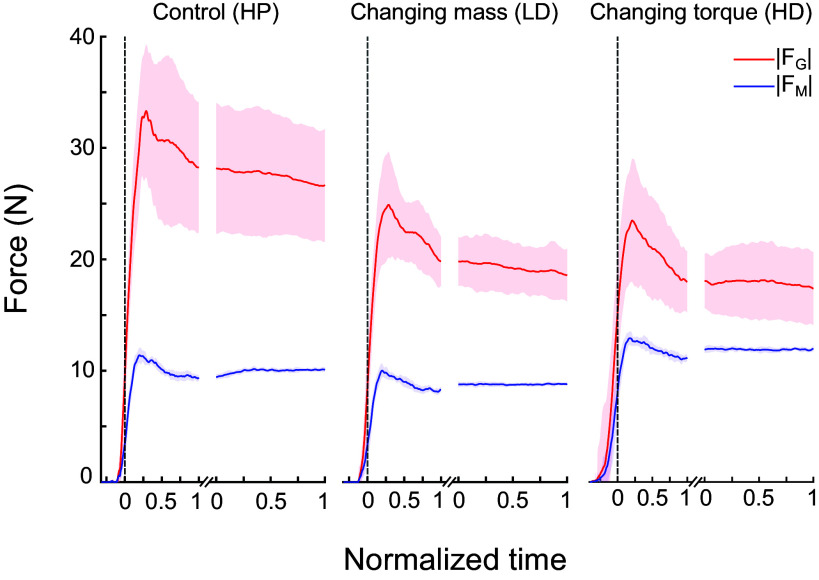
Temporal evolution of manipulation and grasp forces. Time course of grasp and manipulation forces (*F*_G_ and *F*_M_, respectively), averaged across 17 trials (*trials 4–20*) and all subjects, is shown for each experimental condition performed with the right center of mass (CM) (shaded areas denote standard error of the mean). Before averaging across trials, data were time normalized from lift onset to the end of lift and for the one-second epoch of object hold (these two epochs are separated by a break in the *x*-axis in each plot). The vertical dashed line denotes object lift onset. HD, heavy mass, distal mass position; HP, heavy mass, proximal mass position; LD, light mass, distal mass position.

Figure 4 shows the time course of *F*_G_ and *F*_M_ averaged across all subjects for each experimental condition. These data reveal that *F*_G_ was characterized by greater across-subject variability than *F*_M_, which is expected given the well-known idiosyncratic nature of *F*_G_. During object hold, *F*_G_ was larger for the heavy than light mass condition (HP and LD, respectively) but smallest for the large torque condition (HD). In contrast, *F*_M_ was largest for the HD condition and only slightly larger for the HP than for the LD condition. We report the results of the analysis of *F*_G_ and *F*_M_ as a function of object properties.

### Manipulation Force Is Sensitive to Changes in Object Mass and Torque

**Figure 5.**
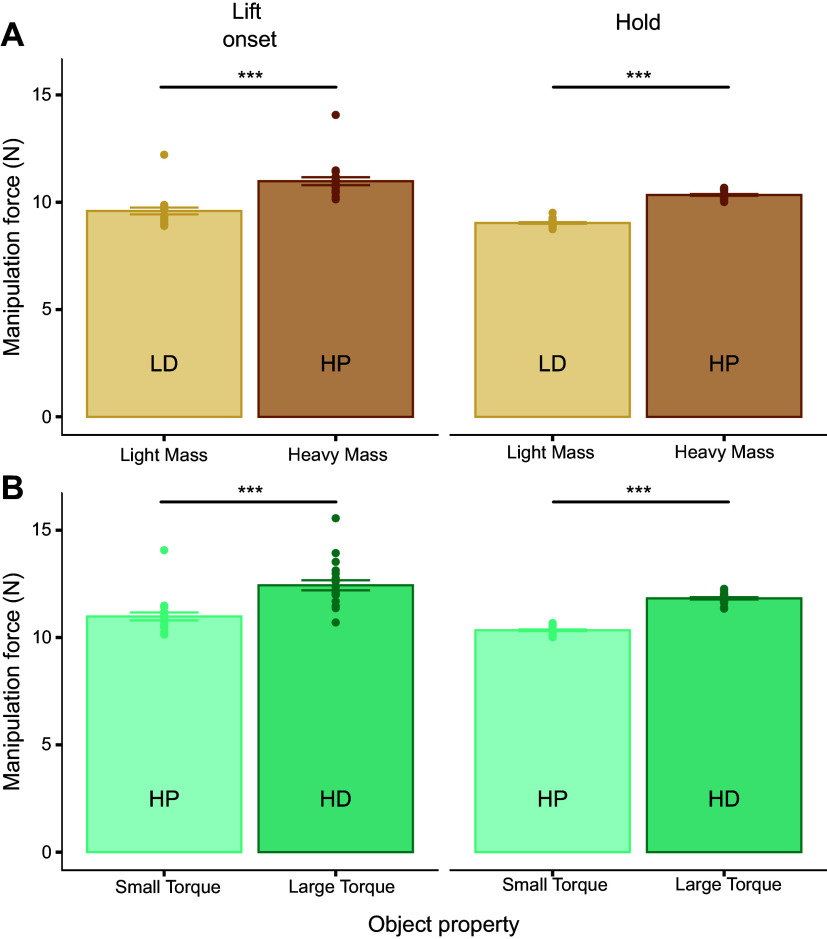
Manipulation force modulation as a function of changes in object mass and torque. *A* and *B*: manipulation force (*F*_M_) measured at object lift onset and hold as a function of object mass [light mass, distal mass position (LD) vs. heavy mass, proximal mass position (HP)] and torque [HP vs. heavy mass, distal mass position (HD)], respectively. Bars are data averaged across all subjects. Filled symbols within each experimental condition denote data from individual subjects (vertical bars denote the standard error of the mean). ***Statistically significant differences (*P* < 0.0001).

*F*_M_ modulation was sensitive to changes in both object mass and torque at lift onset (*t*_19.10_ = −16.13, *P* < 0.0001; *t*_18.97 _= 10.32, *P* < 0.0001, respectively) and hold (*t*_18.75_ = −43.66, *P* < 0.0001; *t*_19.01 _= 37.34, *P* < 0.0001, respectively) (Fig. 5). Specifically, *F*_M_ increased with increasing mass and torque (Fig. 5, *A* and *B*, respectively).

### Sensitivity of Manipulation Force Components to Changes in Object Mass and Torque

To further investigate the modulation of *F*_M_ to object mass and torque, we analyzed the modulation of its tangential and normal components (*^y^F*_M_ and *^z^F*_M_, respectively). The rationale for this analysis was that the role of *F*_M_ for controlling object pose (position and orientation) can be further decomposed into two functional roles denoted by its components: *^y^F*_M_ contributes to the control of object pose which, for our task, consists primarily of object vertical lift and orientation, whereas *^z^F*_M_ contributes only to changes in object orientation.

**Figure 6.**
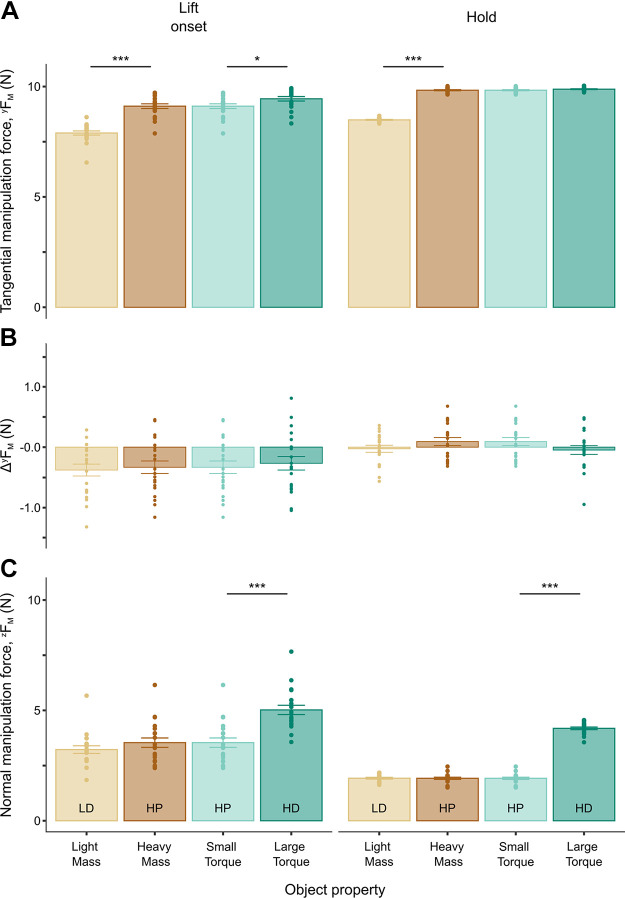
Modulation of manipulation force components as a function of changes in object mass and torque. *A* and *C*: the tangential and normal *F*_M_ components, respectively, as a function of object mass and torque at object lift onset and hold. *B*: the difference between the index finger and thumb tangential *^y^F*_M_ component (Δ*^y^F*_M_). Data are plotted in the same format as Fig. 5. *,***Statistically significant differences (*P* < 0.01 and 0.0001, respectively). HD, heavy mass, distal mass position; HP, heavy mass, proximal mass position; LD, light mass, distal mass position.

We found that *^y^F*_M_ significantly increased with increasing object mass at lift onset and hold (*t*_19.11_ = −17.88, *P* < 0.0001; *t*_19.05_ = −67.80, *P* < 0.0001, respectively). In contrast, *^y^F*_M_ was insensitive to changes in object torque at hold (*t*_32.06 _= 2.54, *P* > 0.01), but increased with increasing object torque at lift onset (*t*_19.04 _= 3.50, *P* = 0.00238) (Fig. 6*A*). However, *^z^F*_M_ significantly increased only when object torque increased (*t*_18.96 _= 14.30, *P* < 0.0001; *t*_19.04 _= 62.44, *P* < 0.0001, for lift onset and hold, respectively) (Fig. 6*C*), whereas it was insensitive to changes in object mass (*t*_18.98_ = −2.74, *P* > 0.01; *t*_19.02 _= 0.16, *P* > 0.5, for lift onset and hold, respectively) (Fig. 6*C*).

We should note that, in our task, *^y^F*_M_ can contribute to controlling both object position (primarily vertical lift) and orientation (object tilt along the frontal plane). The aforementioned results show that *^y^F*_M_ was indeed modulated to both mass and torque, even though this was only found at object lift onset. However, the dual function of *^y^F*_M_ is better captured by the difference between the index and thumb finger’s *^y^F*_M_, Δ*^y^F*_M_, than the magnitude of the *^y^F*_M_ vector. This is because Δ*^y^F*_M_ removes the *^y^F*_M_ contribution to object position control, and therefore is the only portion of *^y^F*_M_ that contributes to object orientation control (*Eq. 2*). We found that Δ*^y^F*_M_ was not modulated at either epoch as a function of object mass (*t*_215.36 _= 0.29, *P* > 0.5; *t*_37.57_ = −2.67, *P* > 0.01, for lift onset and hold, respectively) or torque (*t*_554.63_ = −0.10, *P* > 0.5; *t*_38.26 _= 0.74, *P* > 0.1, for lift onset and hold, respectively) (Fig. 6*B*). By combining the results in Fig. 6, *A* and *B*, we conclude that the modulation of *^y^F*_M_ at lift onset as a function of torque corresponds to the portion of *^y^F*_M_ responsible for position control. Therefore, we confirmed that the contribution of the tangential component of *F*_M_ to object orientation control was negligible. Furthermore, *F*_M_ components (Fig. 6), rather than *F*_M_ as a whole (Fig. 5), were uniquely sensitive to changes in object mass or torque in ways that closely reflect their distinct functional role.

### Grasp Force and Excess Grasp Force Are Sensitive to Changes in Object Mass and Torque

**Figure 7.**
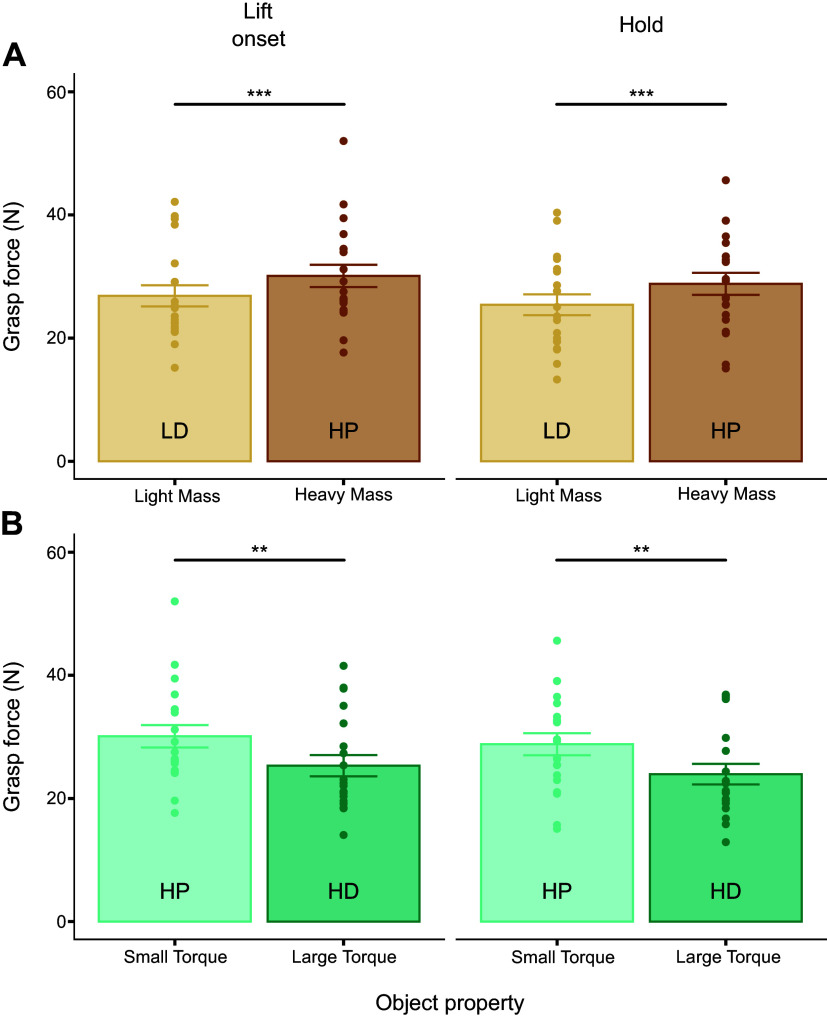
Grasp force modulation as a function of changes in object mass and torque. *A* and *B*: grasp force (*F*_G_) measured at object lift onset and hold as a function of object mass [light mass, distal mass position (LD) vs. heavy mass, proximal mass position (HP)] and torque [HP vs. heavy mass, distal mass position (HD)], respectively. **,***Statistically significant differences at the *P* < 0.001 and *P* < 0.0001 levels, respectively. Data are plotted in the same format as Fig. 5.

*F*_G_, like *F*_M_, was also sensitive to changes in object mass and object torque. Specifically, at both object lift onset and hold, *F*_G_ significantly increased with increasing object mass (*t*_18.98_ = −4.16, *P* < 0.0001; *t*_19.05_ = −5.92, *P* < 0.0001, respectively) (Fig. 7*A*) and decreased with increasing object torque (*t*_19.00_ = −4.29, *P* < 0.001; *t*_18.99_ = −4.05, *P* < 0.001, respectively) (Fig. 7*B*). Visual comparison of Figs. 5 and 7 reveals larger between-subject variability at both task epochs for *F*_G_ than *F*_M_.

We note that *F*_G_ consists of two components: the minimally required grasp force to prevent object slip ($F_{\text{G}}^{\min}$) and the difference between *F*_G_ and $F_{\text{G}}^{\min}$, denoted as excess grasp force ($F_{\text{G}}^{\text{EX}}$), i.e., grasp force that is above what is required to prevent object slip (see methods). Distinguishing between these two components is important because $F_{\text{G}}^{\min}$ is biomechanically obligatory, whereas $F_{\text{G}}^{\text{EX}}$ is not and, in turn, reflects idiosyncratic control strategies in response to perceived changes in the risk of object slip. Therefore, the following analysis focused on the modulation of $F_{\text{G}}^{\text{EX}}$ to changes in object mass and torque.

**Figure 8.**
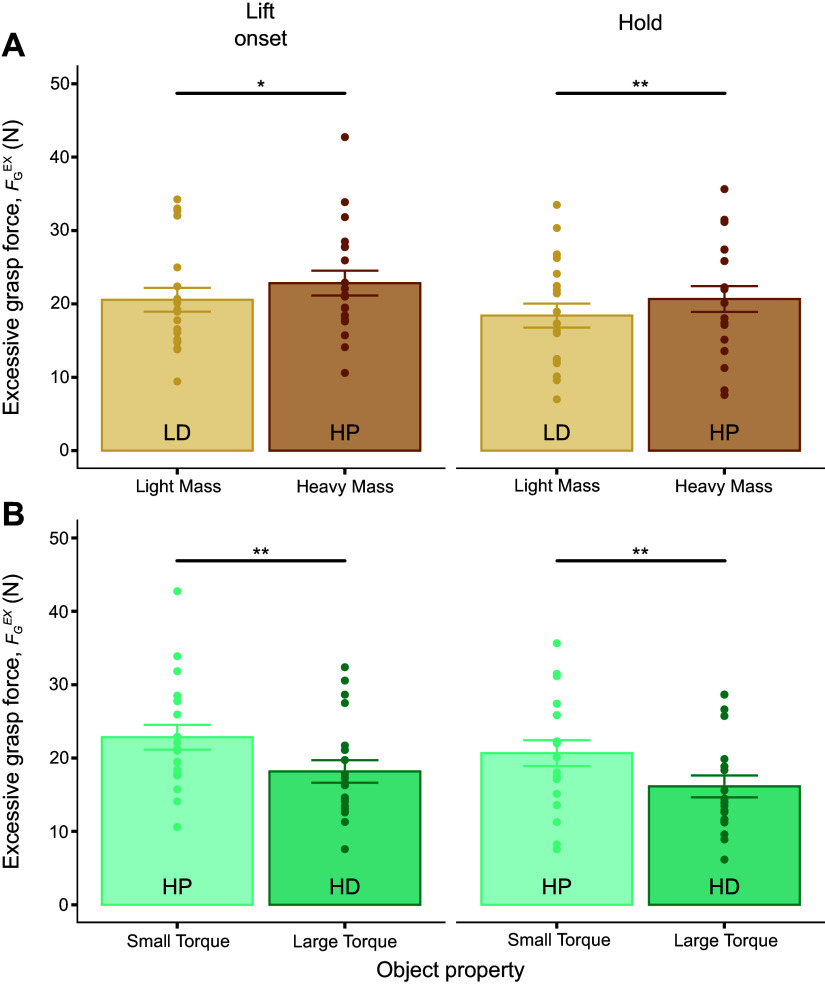
Excessive grasp force modulation as a function of changes in object mass and torque. *A* and *B*: excessive grasp force ($F_{\text{G}}^{\text{EX}}$) measured at object lift onset and hold as a function of object mass [light mass, distal mass position (LD) vs. heavy mass, proximal mass position (HP)] and torque [HP vs. heavy mass, distal mass position (HD)], respectively. *,**Statistically significant differences at the *P* < 0.01 and *P* < 0.001 levels, respectively. Data are plotted in the same format as Fig. 5.

$F_{\text{G}}^{\text{EX}}$ modulation exhibited the same trend described for *F*_G_ (Fig. 8): subjects responded to increasing object mass by exerting greater $F_{\text{G}}^{\text{EX}}$ at lift onset and hold (Fig. 8*A*) despite the fact that this modulation is functionally unnecessary to minimize object slip (*t*_18.98_ = −3.11, *P* < 0.01; *t*_19.02_ = −3.89, *P* < 0.001, respectively). As described for *F*_G_ modulation, subjects responded to increasing object torque by decreasing $F_{\text{G}}^{\text{EX}}$ (*t*_19.00_ = −4.60, *P* < 0.001; *t*_18.99_ = −3.90, *P* < 0.001, for lift and hold, respectively) (Fig. 8*B*). These results were found at both object lift onset and hold.

To determine the factors that might have caused the reduction of $F_{\text{G}}^{\text{EX}}$ with increasing torque, we analyzed the contribution of potential changes in friction, object velocity, and/or the vertical distance between thumb and index fingertip to the decrease in $F_{\text{G}}^{\text{EX}}$. We focused on these three factors because they have been implicated with grip force modulation (21). If friction had increased throughout the experiment, subjects could have chosen to exert smaller $F_{\text{G}}^{\text{EX}}$. Similarly, subjects might have responded to a larger object torque by lifting the object more slowly, and the lower load force associated with this behavior would have led to a reduction in $F_{\text{G}}^{\text{EX}}$. Finally, larger vertical distances between thumb and index fingertip would have increased the moment arm of thumb and index finger normal *F*_M_ components, *^y^r* (*Eq. 2*, methods). In turn, the larger *^y^r* could have prompted subjects to exert smaller *^y^F*_M_, thus leading to a decrease in $F_{\text{G}}^{\min}$. Therefore, a reduction in $F_{\text{G}}^{\text{EX}}$ would be expected if $F_{\text{G}}^{\min}$ significantly decreased with increasing *^y^r*.

By comparing object release trials at the beginning versus the end of each experimental session, we found that the coefficient of friction did not change throughout the experimental session (*t* test, *t*_118_ = 0.59, *P* > 0.10). Analysis of object vertical velocity further revealed that subjects did not modulate lifting velocity within either mass or torque experimental condition (*t*_18.97_ = −1.17, *P* > 0.10; *t*_18.98_ = −0.12, *P* > 0.10, respectively). Although we found that the vertical distance between the thumb and index fingertip significantly increased with increasing object torque (*t*_19.00 _= 14.74, *P* < 0.0001), $F_{\text{G}}^{\min}$ did not change significantly (*t*_18.96_ = −1.15, *P* > 0.05) (Supplemental Fig. S2). These results indicate that the reduction in $F_{\text{G}}^{\text{EX}}$ with increasing torque was biomechanically nonobligatory.

### Digit Force Vector Angles as a Function of Object Property

**Figure 9.**
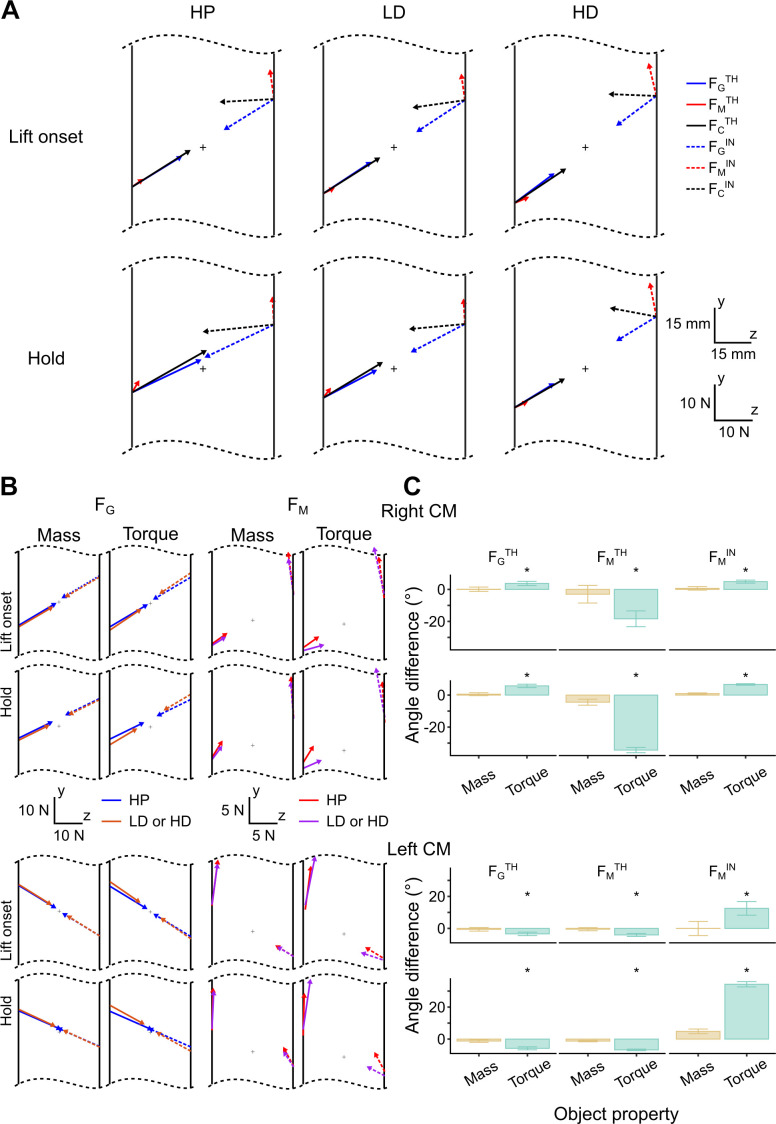
*F*_G_ and *F*_M_ vectors and vector angles as a function of changes in object mass and torque at object lift onset and hold. *A*: each of the six diagrams denotes the object and the vector of thumb and index finger forces applied at object lift onset and hold (*top and bottom* row, respectively). Data are averages of *trials 4*–*20* from one subject (S5). The three experimental conditions [heavy mass, proximal mass position (HP), light mass, distal mass position (LD), and heavy mass, distal mass position (HD) of R center of mass (CM)] are shown in each column. In each diagram, the digit force vectors and their points of application are shown for thumb and index finger contact force ($F_{\text{C}}^{\text{TH}}$ and $F_{\text{C}}^{\text{IN}}$, respectively; black solid and dotted lines), grasp force ($F_{\text{G}}^{\text{TH}}$ and $F_{\text{G}}^{\text{IN}}$, respectively; blue solid and dotted lines), and manipulation force ($F_{\text{M}}^{\text{TH}}$ and $F_{\text{M}}^{\text{IN}}$, respectively; red solid and dotted lines). The “+” denotes the geometric center of the handle. *B*: digit force vectors associated with the R CM (*top two rows*) and L CM (*bottom two rows*) at task epochs (lift onset and hold). For each row, the left two plots show thumb and index finger *F*_G_ vectors (blue solid and dashed lines, respectively), whereas the right two plots show thumb and index finger *F*_M_ vectors (red solid and dashed lines, respectively) from the HP condition. Orange and purple lines denote *F*_G_ and *F*_M_, respectively, from conditions where object mass or torque was changed (LD and HD, respectively). As the magnitude of the *F*_G_ vector is larger than *F*_M_, these forces are plotted using different ranges of calibration bars (*top*). *C*: angle differences of mass- and torque-changing comparisons (mass: LD minus HP; torque: HD minus HP) for the *F*_G_ vector (thumb) and *F*_M_ vector (thumb and index finger) at object lift onset and hold (*top* and *bottom rows*, respectively) with right and left CM conditions. Error bars denote angular standard errors. *Within-condition differences (light to heavy mass; small to large torque) in force vector angles. Data in *B* and *C* are averages of all subjects.

Figure 9*A* shows the digit force vectors of thumb and index fingers for each experimental condition (columns) measured and computed at object lift onset and hold (top and bottom row, respectively) for the R CM condition of a representative subject. Our algorithm decomposes each digit contact force, *F*_C_ (black solid and dotted lines, respectively) into *F*_G_ and *F*_M_. We note that the thumb and index *F*_G_ vectors ($F_{\text{G}}^{\text{TH}}$ and $F_{\text{G}}^{\text{IN}}$, blue solid and dotted lines, respectively) cancel each other out, as *F*_G_ is only responsible for object slip prevention rather than changing object pose. In contrast, thumb and index finger *F*_M_ vectors (red solid and dotted lines, respectively) are responsible for changing object pose. Therefore, the net effect of thumb and index finger *F*_M_ consists of lifting the object and preventing object tilt. Note that *F*_G_ vector angle barely changes when changing mass (HP vs. LD) and increases slightly when changing torque (HP vs. HD). Furthermore, the *F*_G_ vector angle undergoes minimal changes from lift onset to hold. In contrast, the *F*_M_ vector angle changed drastically when torque increased, but remained relatively unchanged when mass increased.

Figure 9*B* shows the mean force vectors of the thumb and index fingers for *F*_G_ and *F*_M_ at lift onset and hold. The first and third columns showcase conditions of different mass and the same torque, whereas the second and fourth columns showcase conditions of different torque and the same mass. As the resultant *F*_G_ force summed from thumb and index fingers equals zero, $F_{\text{G}}^{\text{TH}}$ and $F_{\text{G}}^{\text{IN}}$ vector directions change in the same way in both mass and torque changes. In contrast, $F_{\text{M}}^{\text{TH}}$ and $F_{\text{M}}^{\text{IN}}$ are context dependent as *F*_M_ dominates object dynamics. Finally, *F*_M_ exhibited large changes from object lift onset to hold, whereas *F*_G_ had marginal changes throughout the experimental task.

Figure 9*C* shows the mean difference in the angles of the $F_{\text{G}}^{\text{TH}}$, $F_{\text{M}}^{\text{TH}}$, and $F_{\text{M}}^{\text{IN}}$ vectors as a function of object mass (LD minus HP) and torque (HD minus HP) for both CM conditions and task epochs. Changing mass did not cause statistically significant changes in force vectors across all experimental conditions (see lower and upper bound differences in Table 1). In contrast, changing object torque caused statistically significant differences for both *F*_G_ and *F*_M_ across all conditions (Table 1). We note that changing object torque (Fig. 9*B*, columns 2 and 4) shows a larger angular difference in both *F*_G_ and *F*_M_ vectors. Furthermore, *F*_M_ vectors’ angular difference due to changes in mass or torque is much larger than *F*_G_’s angular difference in both task epochs.

**Table 1.** Circular statistics: posterior density intervals of F_G_ and F_M_ vector angles as a function of object property at object lift onset and hold

Vector	Comparison			HPD Interval Lower Bound, °	HPD Interval Upper Bound, °
	CM	Epoch	Object Property		
$F_{\text{G}}^{\text{TH}}$	R	Lift	HP	28.454	32.386
			LD	28.658	32.611
			HD	33.852	37.586
		Hold	HP	24.957	27.964
			LD	25.135	28.126
			HD	29.517	32.411
	L	Lift	HP	−33.432	−29.093
			LD	−34.126	−29.876
			HD	−37.506	−33.738
		Hold	HP	−26.174	−22.549
			LD	−27.129	−23.686
			HD	−30.639	−27.478
$F_{\text{M}}^{\text{TH}}$	R	Lift	HP	39.831	49.406
			LD	34.009	43.918
			HD	9.375	15.831
		Hold	HP	52.709	57.747
			LD	49.200	54.439
			HD	26.067	30.730
	L	Lift	HP	82.783	84.715
			LD	81.885	83.703
			HD	77.188	79.168
		Hold	HP	87.216	88.608
			LD	86.262	87.637
			HD	81.625	83.129
$F_{\text{M}}^{\text{IN}}$	R	Lift	HP	82.271	84.520
			LD	81.572	83.699
			HD	75.914	77.693
		Hold	HP	85.132	86.638
			LD	84.460	85.977
			HD	79.902	81.311
	L	Lift	HP	35.642	47.339
			LD	32.999	43.740
			HD	11.724	19.821
		Hold	HP	55.053	61.712
			LD	51.503	58.013
			HD	29.032	35.306

The lower and upper boundary (95% highest posterior density interval) of force vector angles (°) computed by circular statistics are shown for thumb grasp force, thumb manipulation force, and index finger manipulation force ($F_{\text{G}}^{\text{TH}}$, $F_{\text{M}}^{\text{TH}}$ and $F_{\text{M}}^{\text{IN}}$, respectively). For each force vector, overlapping intervals denote no statistically significant differences between force vector angles for within-condition comparisons [effect of object mass: heavy mass, proximal mass position (HP) vs. light mass, distal mass position (LD); effect of object torque: HP vs. heavy mass, distal mass position (HD)], and vice versa for nonoverlapping intervals. Mean force vector angles and significant differences are shown in Fig. 9. See text for details on the circular mixed effect model design. CM, center of mass; HPD, highest posterior density.

## DISCUSSION

**Figure 10.**
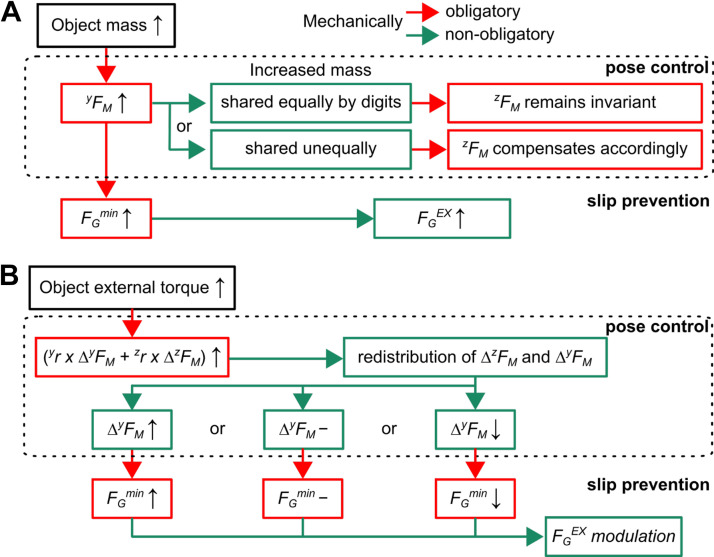
Chain of effects caused by changes in object properties on the coordination of grasp and manipulation forces. The two diagrams summarize the chain of effects caused by changes in object properties on grasp and manipulation forces (*F*_G_ and *F*_M_, respectively). Upward and downward arrows within boxes denote an increase or decrease of a given variable, respectively, whereas horizontal lines denote no change. Red and green lines denote biomechanically obligatory and nonobligatory effects, respectively. *A*: when the object mass increased but the external torque was held constant, the tangential component of manipulation force (*^y^F*_M_) obligatorily increased and led to a nonobligatory increase in excessive grasp force ($F_{\text{G}}^{\text{EX}}$). Subjects can choose how to share *^y^F*_M_ between thumb and index fingertip, with each type of sharing resulting in different effects on the magnitude of the normal component of *F*_M_ (^z^*F*_M_). *B*: the increase in the object’s external torque but with the mass held constant resulted in an obligatory modulation of *F*_M_, which caused a redistribution of normal and tangential *F*_M_ components. Note that this redistribution is mathematically indeterminate, i.e., subjects can choose to modulate the difference between thumb and index fingertip’s tangential *F*_M_ components (Δ*^y^F*_M_) in three different ways. However, each Δ*^y^F*_M_ solution leads to different changes in the minimally required grasp force ($F_{\text{G}}^{\text{min}}$). Regardless of the specific $F_{\text{G}}^{\text{min}}$ change induced by Δ*^y^F*_M_ modulation, subjects modulated $F_{\text{G}}^{\text{EX}}$ based on their idiosyncratic preferences and perceived risk of object slip.

Decomposing human digit forces into grasp and manipulation forces (*F*_G_ and *F*_M_, respectively) using MGFD effectively identifies the distinct contributions of digit force components responsible for preventing object slip from those involved with object pose control. As we have shown in Fig. 1, the MGFD outperforms the conventional digit force analyses of normal and tangential forces for nonzero digit offset and/or when the object rolls (Fig. 1). By separating biomechanically obligatory from nonobligatory effects (Fig. 10), analysis of *F*_G_ and *F*_M_ allowed us to determine the strategy that subjects chose to respond to changes in object properties—mass or external torque. The main finding of the present work is that *F*_G_ and *F*_M_ were both sensitive to changes in each object property. However, further analysis revealed that the *F*_M_ component responsible for object orientation control (^z^*F*_M_) was only sensitive to changes in object torque. In contrast, *F*_G_ was distinctly modulated to changes in object mass and torque. This distinct sensitivity exhibited by *F*_G_ and *F*_M_ modulation to changes in object properties likely underscores differences in their functional role and, possibly, sensorimotor control mechanisms. Furthermore, we confirmed our previous finding (20) by showing that *F*_G_ and *F*_M_ are controlled in a feedforward and feedback fashion, respectively. In the following section, we discuss our results in the context of control strategies versus biomechanically obligatory effects, as well as putative underlying neural mechanisms.

### Pose Control Requirements Induced by Changes in Mass versus External Torque

Manipulation force (*F*_M_) determines changes in both object position and orientation. In the present study, grasp surfaces were parallel to each other and perpendicular to the direction of gravity. Therefore, modulation of the tangential and normal components of *F*_M_ (*^y^F*_M_ and *^z^F*_M_, respectively) dictates the extent to which they affect changes in vertical and horizontal object position, respectively. In contrast, both *F*_M_ components could theoretically contribute to changes in object orientation. Our results indicate that *^y^F*_M_ and *^z^F*_M_ were differentially sensitive to changes in object properties that affected either its vertical position (increase in mass; Fig. 6*A*) or orientation control (increase in torque; Fig. 6*C*), respectively. As done in our previous work (20), we separated biomechanically obligatory from nonobligatory effects by analyzing the chain of effects caused by changing object properties (Fig. 10).

Increasing object mass while keeping the external torque constant causes an obligatory increase in the tangential component of *F*_M_ responsible for changing object position, *^y^F*_M_ (Fig. 10*A*). However, this modulation can occur through indeterminate patterns of *^y^F*_M_ sharing between the digits. Importantly, how *^y^F*_M_ is shared impacts obligatory changes in *^z^F*_M_ such that it either remains invariant for equal *^y^F*_M_ sharing or is modulated to compensate for the unequal *^y^F*_M_ sharing. As the compensatory torque requirement is invariant with increasing object mass, the equal *^y^F*_M_ sharing does not cause an additional torque and, therefore, *^z^F*_M_ can remain invariant. In contrast, if *^y^F*_M_ is shared unequally between the digits in response to a larger object mass, the additional torque generated by *^y^F*_M_ must be compensated for by *^z^F*_M_ modulation. We found that subjects adopted an equal sharing of *^y^F*_M_ as a function of object mass (Fig. 6*B*). This finding could be interpreted as a strategy aiming at simplifying object pose control as it avoids generating additional torques that would have to be compensated for.

Increasing object torque requires subjects to generate a greater compensatory torque to maintain a given object orientation, this effect being biomechanically obligatory (Fig. 10*B*). As noted earlier, *^y^F*_M_ and *^z^F*_M_ must covary to generate the desired compensatory torque (for clarity, Fig. 10*B* shows only *^y^F*_M_). However, their distribution between the digits (Δ*^y^F*_M_) is indeterminate (we describe the effects of *^y^F*_M_ distribution on *F*_G_ in *Object Slip Prevention Requirements Caused by Changes in Mass versus in External Torque*). We found that, at object lift onset, *^y^F*_M_ magnitude increased with both object mass and torque whereas, during object hold, *^y^F*_M_ magnitude was modulated only to object mass (Fig. 6*A*). Importantly, however, we found that the difference between the index and thumb finger’s *^y^F*_M_ (Δ*^y^F*_M_), i.e., the portion of *^y^F*_M_ responsible for object orientation control, was not modulated as a function of object torque at both task epochs (Fig. 6*B*). Thus, object orientation was entirely controlled by selective modulation of *^z^F*_M_ to object torque (Fig. 6*C*). This finding suggests that rather than generating the compensatory torque by modulating both *F*_M_ components, participants modulated only *^z^F*_M_ to compensate for the increase in external torque. The modulation of *^z^F*_M_ to object torque in the grasp plane could have been mediated by fast-adapting type-I mechanoreceptive afferents (FA-I), as well as slowly adapting type I and -II afferents (SA-I and SA-II, respectively), whose response has been shown to modulate with torque magnitude in humans (tangential torque, 34).

From a functional perspective, the selective modulation of *^z^F*_M_ to changes in object torque, but not mass, supports our hypothesis of selective modulation of *F*_M_ to factors affecting object pose control, but not object slip risk. Note that the moment arm of *^y^F*_M_, i.e., object width (59.2 mm), was always substantially larger than the moment arm of *^z^F*_M_, i.e., digit vertical offset (48.8 mm). As a result, to generate the same torque, the smaller *^z^F*_M_ moment arm requires larger forces than *^y^F*_M_ acting on its larger moment arm. Therefore, modulation of *^z^F*_M_ only in response to changes in external torque suggests that participants did not prioritize minimization of overall manipulation force. In the following section, we discuss the effects of changing object properties on grasp force.

### Object Slip Prevention Requirements Caused by Changes in Mass versus in External Torque

Grasp force (*F*_G_) is responsible for preventing object slip in response to any perturbation, i.e., grasp stability. *F*_G_ increased with increasing object mass (Fig. 7*A*), which suggests that subjects perceived the increase in mass as a larger risk of object slip. This finding is partially consistent with our hypothesis, as *F*_G_ was also modulated to changes in object torque. We note that to prevent the object from slipping, subjects must exert *F*_G_ with a magnitude at least equal to the minimum grasp force ($F_{\text{G}}^{\text{min}}$), this being a biomechanically obligatory phenomenon. Therefore, *F*_G_ above $F_{\text{G}}^{\text{min}}$, which we denoted as excessive grasp force ($F_{\text{G}}^{\text{EX}}$), is nonobligatory and reflects idiosyncratic preferences aimed at ensuring grasp stability. We found that $F_{\text{G}}^{\text{EX}}$ also increased with increasing object mass (Fig. 8*A*). As done earlier for *F*_M_, we analyzed the extent to which this effect was biomechanically obligatory or nonobligatory (Fig. 10).

Increasing object mass increases the risk of object slip and, as mentioned previously, results in an obligatory increase in *^y^F*_M_ which, in turn, causes an obligatory increase in $F_{\text{G}}^{\text{min}}$ (Fig. 10*A* and Supplemental Fig. S2*A*). As $F_{\text{G}}^{\text{EX}}$ also increased with mass, the question arises as to whether $F_{\text{G}}^{\text{EX}}$ increased proportionally with $F_{\text{G}}^{\text{min}}$. If found, such a relation would imply that subjects perceived the change in $F_{\text{G}}^{\text{min}}$, to which they would have responded by increasing $F_{\text{G}}^{\text{EX}}$. This behavior would be consistent with previous work reporting scaling of grip force with increasing minimally required grip force (11, 35–38, for review, see Refs. 39 and 40). Although subjects responded to larger $F_{\text{G}}^{\text{min}}$ by increasing $F_{\text{G}}^{\text{EX}}$, the linear relationship between these two variables was very weak (*r*^2^ values measured at object lift onset and hold were both <0.15). Therefore, although we cannot exclude that $F_{\text{G}}^{\text{EX}}$ modulation occurred in response to an increase in $F_{\text{G}}^{\text{min}}$, such response would appear to be disproportionately large. We interpret this phenomenon as evidence for subjects prioritizing grasp stability over minimizing digit forces, this finding being consistent with our previous work on the effect of grip configuration on *F*_G_ and *F*_M_ (20).

Increasing object external torque requires subjects to generate a larger compensatory torque (*Eq. 2*, methods) to maintain the desired object orientation throughout the manipulation. This biomechanically obligatory response results in the earlier-described effects on *F*_M_ components (Fig. 10*B*) which, in turn, affect $F_{\text{G}}^{\text{min}}$ in three possible ways: it can increase, remain invariant, or decrease. We found that, although *^y^F*_M_ and $F_{\text{G}}^{\text{min}}$ remained invariant when increasing external torque (Fig. 6*A* and Supplemental Fig. S2*B*, respectively), $F_{\text{G}}^{\text{EX}}$ decreased (Fig. 8). Together, these observations indicate that, although idiosyncratic, $F_{\text{G}}^{\text{EX}}$ was sensitive to changes in object torque. An increase in friction at the digit-grasp surface and/or a decrease in object velocity could have accounted for the decrease in $F_{\text{G}}^{\text{EX}}$ with increasing object torque. However, neither of these variables was significantly modulated as a function of object torque. Similarly, the modulation of vertical distance between thumb and index fingertip could not account for the decrease in $F_{\text{G}}^{\text{EX}}$, as this did not lead to a reduction in $F_{\text{G}}^{\text{min}}$. We note that previous studies have reported grip force increases when increasing tangential torque requirements of precision grips (41, 42) and increasing object instability of five-digit grasps (43). The discrepancy between this previous work and our findings is likely due to several methodological differences, including the use of grip force instead of *F*_G_, collinear versus noncollinear digit contacts, the compensatory torque direction (tangential torque vs. torque within the grasp plane, 41, 42), and the number of digits engaged in the grasp (two vs. five, 43).

As we could rule out a decrease in $F_{\text{G}}^{\text{min}}$ as the main driver of the decrease in $F_{\text{G}}^{\text{EX}}$ (Supplemental Fig. S2*B*), we interpret $F_{\text{G}}^{\text{EX}}$ modulation as reflecting the misperception of a lower risk of object slip in response to larger torque. As noted earlier, the response of FA-I, SA-I, and SA-II is modulated to torque magnitude (34). We speculate that the integration of these responses with mechanoreceptor responses arising from holding an object with a constant mass was suboptimal, as the subjects erroneously interpreted these signals as a reduced risk of object slip. It is worth noting that Loutit et al. (34) found that the torque sensitivity of most afferent types decreased with larger background normal forces. Therefore, one could speculate that subjects might have decreased $F_{\text{G}}^{\text{EX}}$ with increasing torque to optimize the mechanoreceptors’ sensitivity to changes in torque. However, further work is needed to test these interpretations.

### Grasp and Manipulation Forces: Differential Sensitivity to Object Properties and Control Mechanisms

Our analyses revealed that participants responded to changes in torque by increasing the *F*_M_ component contributing to object orientation control (^z^*F*_M_), whereas changing object mass caused the *F*_M_ component contributing to object vertical position (^y^*F*_M_) to increase. In contrast, *F*_G_ was modulated to both changes in object mass and torque. Therefore, *F*_M_ and *F*_G_ were differentially sensitive to changes in object properties, even though *F*_M_ modulation was more selective than *F*_G_. Another important observation is that we again found that *F*_M_ and *F*_G_ are mediated by different sensorimotor control mechanisms (20, Supplemental Fig. S1). Specifically, the magnitude and angle of *F*_M_ underwent significant modulation from object lift onset to hold, indicating that subjects are unable to accurately predict the dynamics of the object during the lift. In contrast, subjects did not significantly modulate *F*_G_ when transitioning from object lift onset to hold. Together, these observations provide novel insights into how the CNS may coordinate two functionally distinct components of digit forces for dexterous object manipulation. The different tolerance for errors in object pose control and object slip prevention might play a critical role in the earlier-described distinct modulation of *F*_M_ and *F*_G_ to changes in object properties.

The control of object pose in three-dimensional space is characterized by three positional and three rotational constraints when manipulating an object with two soft-contact digits. Therefore, successful manipulation requires the coordination of a total of eight variables for which there exist multiple digit force component combinations—rather than a unique solution—that can control object pose equally well. Nevertheless, a deviation from any of these digit force combinations directly results in an error in object pose, which in our task consists of object roll. Therefore, online feedback information is critically important to accurately modulate *F*_M_ and dynamically control object pose. Tactile afferents are likely the predominant source of inputs for monitoring the transition from grasp to manipulation, as well as the dynamic phase of manipulation. In contrast, visual feedback, due to its longer sensorimotor latency, may play a more critical role in the early phase of sensorimotor adaptation, i.e., the first three trials in our task as subjects learn to modulate digit forces to attain the desired compensatory torque at object lift onset and throughout the manipulation.

Unlike the feedback-mediated control of *F*_M_, any value above $F_{\text{G}}^{\text{min}}$ can prevent object slip. Unless there are additional task demands on *F*_G_, e.g., ensuring grasp stability to resist perturbations in an unstructured environment, anticipatory modulation of *F*_G_ is sufficient for successfully preventing object slip. The earlier-described prioritization of grasp stability over efficient digit force control is somewhat puzzling, given that no perturbations in object mass or torque were presented within blocks of trials. Further work is needed to test whether grasp stability prioritization is a default strategy regardless of task constraints, e.g., predictability of object properties and/or accuracy demands.

### Role of Tactile Afferents for the Control of Grasp and Manipulation Forces

The modulation of *F*_G_ and *F*_M_ to object properties and during the transition from object lift onset to hold point to the involvement of sensorimotor integration mechanisms is based on tactile mechanoreceptors. The work by Birznieks et al. (44) revealed that SA-I, SA-II, and FA-I tactile afferents are broadly tuned to a preferred force direction. This phenomenon suggests that the combined output of tactile mechanoreceptors could be used by the CNS to decode the direction of the fingertip force vector. A subsequent study further revealed that the activity of a population of tactile afferents encodes grip stability before slips occur, suggesting that grip force could be modulated by sensory signals indicating skin-object contact safety, thus prompting grip force adjustments to prevent slips (45). Psychophysical evidence has also shown that human participants can use normal skin indentation and tangential skin stretch to perceive the magnitude of contact pressure (46). Regarding the modulation of digit forces during manipulation, the measurement of fingertip skin deformation patterns has provided significant insights into the phenomena responsible for digit force regulation. This approach allowed measurement of the stick ratio, i.e., the proportion of finger contact that is not slipping, and strain rate, which quantifies the parts of the finger experiencing the largest deformation. Despite changes in friction of the contact surface, Schiltz et al. (47) found that subjects regulated grip force resulting in similar levels of stick ratio and strain rates. These findings suggest that these two variables are critically important for grip force regulation in response to partial object slips. Further insights have been provided by a recent study indicating that fingertip skin strain occurring ∼100 ms before object lift off drives the subsequent modulation of grip force to changes in contact surface friction (48).

We cannot directly compare the results of the aforementioned work with the present study due to methodological differences, e.g., task requirements (object pose control in addition to object slip prevention) and digit force analytical approaches (traditional vs. MGFD). Nevertheless, the aforementioned evidence allows us to speculate that distinct magnitudes (Figs. 5, 6, 7, and 8) and angles (Fig. 9) of *F*_G_ and *F*_M_ vectors associated with different object properties and task epochs could have elicited distinct patterns of tactile inputs. These tactile inputs, driven by distinct skin strain patterns, would have been responsible for online modulation of *F*_M_ throughout object lift and enabling anticipatory control of *F*_G_ before onset of object manipulation. Nevertheless, further work is needed to determine the peripheral sensory representations of *F*_G_ and *F*_M_ and their integration for the control of dexterous manipulation. Ongoing work is addressing the extent to which distinct peripheral motor representations of *F*_G_ and *F*_M_ exist by using surface electromyography of digit and wrist muscles.

### Limitations of the Present Study and Future Research Directions

The ability to decompose three digit forces and one moment of force per digit (8 variables) into two forces, *F*_G_ and *F*_M_, does not necessarily imply that there exist corresponding neural representations at the central and/or peripheral levels of the neuromuscular system, e.g., primary motor cortex and/or hand muscle activity, respectively. Therefore, future work should address the extent to which these representations exist and whether these could be eventually used for biomedical applications, e.g., brain-machine interfaces and controllers for assistive devices.

Another limitation is that we have addressed the sensitivity of *F*_G_ and *F*_M_ in the context of only two object properties. However, it remains to be established whether the differential sensitivity of these forces and distinct sensorimotor mechanisms may also depend on other task characteristics, e.g., object rigidity and precision requirements. Finally, future work should also address the role of multisensory integration (primarily vision and touch) in the coordination of *F*_G_ and *F*_M_ at different stages of sensorimotor adaptation of dexterous manipulation.

## DATA AVAILABILITY

Supplemental materials and all raw data are available on the public access repository, Open Science Framework (https://doi.org/10.17605/OSF.IO/YX2GN) with no restrictions. All data needed to evaluate the conclusions in the paper are present in the paper and/or the Supplementary Materials.

## SUPPLEMENTAL DATA

10.17605/OSF.IO/YX2GN Supplemental Figs. S1 and S2: https://doi.org/10.17605/OSF.IO/YX2GN.

## GRANTS

This work was supported by NIH Grant R21AR081636.

## DISCLOSURES

No conflicts of interest, financial or otherwise, are declared by the authors.

## AUTHOR CONTRIBUTIONS

W.P.N., Y.-H.W., and M.S. conceived and designed research; W.P.N. and Y.-H.W. performed experiments; W.P.N., Y.-H.W., and M.S. analyzed data; W.P.N., Y.-H.W., and M.S. interpreted results of experiments; W.P.N. and Y.W. prepared figures; W.P.N. and Y.W. drafted manuscript; W.P.N., Y.-H.W., and M.S. edited and revised manuscript; W.P.N., Y.-H.W., and M.S. approved final version of manuscript.
